# Epigenetic Alterations in Pediatric Sleep Apnea

**DOI:** 10.3390/ijms22179523

**Published:** 2021-09-01

**Authors:** Emily Catherine Cheung, Matthew Wyatt Kay, Kathryn Jaques Schunke

**Affiliations:** 1 Department of Biomedical Engineering, George Washington University, 800 22nd Street NW, Washington, DC 20052, USA; eccheung@gwmail.gwu.edu (E.C.C.); phymwk@gwu.edu (M.W.K.); 2 Department of Anatomy, Biochemistry & Physiology, Johns A. Burns School of Medicine, University of Hawaii, 651 Ilalo Street, Honolulu, HI 96813, USA

**Keywords:** epigenetic mechanisms of disease, fetal programming, obstructive sleep apnea, DNA methylation, histone modifications, chronic disease

## Abstract

Pediatric obstructive sleep apnea has significant negative effects on health and behavior in childhood including depression, failure to thrive, neurocognitive impairment, and behavioral issues. It is strongly associated with an increased risk for chronic adult disease such as obesity and diabetes, accelerated atherosclerosis, and endothelial dysfunction. Accumulating evidence suggests that adult-onset non-communicable diseases may originate from early life through a process by which an insult applied at a critical developmental window causes long-term effects on the structure or function of an organism. In recent years, there has been increased interest in the role of epigenetic mechanisms in the pathogenesis of adult disease susceptibility. Epigenetic mechanisms that influence adaptive variability include histone modifications, non-coding RNAs, and DNA methylation. This review will highlight what is currently known about the phenotypic associations of epigenetic modifications in pediatric obstructive sleep apnea and will emphasize the importance of epigenetic changes as both modulators of chronic disease and potential therapeutic targets.

## 1. Introduction

Pediatric obstructive sleep apnea (OSA) is a disorder characterized by recurring upper airway obstruction leading to intermittent oxygen desaturations, sustained levels of carbon dioxide, frequent waking during the night, habitual snoring, and excessive daytime sleepiness in children. It is estimated to affect as many as 13% of children between the ages of 3–6 years old, and 2–4% of middle-school age children [1,2]. Infants are particularly vulnerable to obstructive sleep-disordered breathing due to multiple predisposing factors that are often associated with infants, including their small upper airway structure, immature pulmonary mechanics and ventilatory control, high arousal threshold, insensitive laryngeal chemoreflex, and a REM-predominant sleep state [3]. Childhood obesity is also a significant risk factor for OSA [4] and reciprocally, OSA can contribute to obesity [5]. With almost 170 million children currently considered obese, and at least half of those children maintaining obesity into adulthood [6], the number of children with undiagnosed sleep apnea is likely very high. The risk of pediatric OSA also increases in vulnerable populations, such as children from low socioeconomic backgrounds, especially preterm infants [7]. For example, one study found that infants born prior to 32 weeks’ gestation, despite having normal birth weights, exhibit an increased hypoxic ventilatory response indicative of enhanced peripheral chemoreceptor activity, and a higher likelihood of OSA diagnosis later in childhood [8,9].

**Figure 1:**
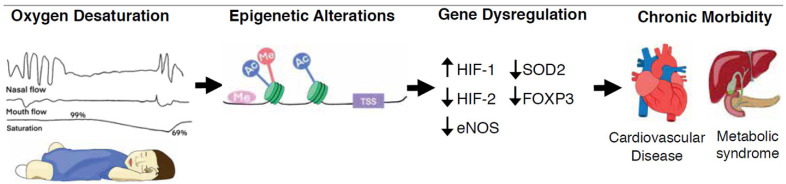
Recurring upper airway obstruction leads to intermittent oxygen desaturations, or hypoxia. Hypoxia-mediated epigenetic mechanisms such as DNA methylation, histone modifications and non-coding RNAs, alter both the chromatin organization and accessibility of genes for transcription factor binding as well as the rates of gene transcription. These heritable, but reversible, epigenetic alterations underly human adaptation or maladaptation to stimuli and may modulate chronic disease. Abbreviations—Me: DNA or histone methylation; TSS: transcription start site; Ac: histone acetylation; HIF-1: hypoxia inducible factor 1; HIF-2: hypoxia inducible factor 2; eNOS: nitric oxide synthase 3; SOD2: superoxide dismutase 2; FOXP3: forkhead box P3; 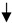: decreased expression; 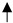: increased expression.

Untreated pediatric OSA often leads to developmental deficits, such as cognitive impairments, attention-deficit/hyperactivity disorder, and poor academic performance [10]. Pediatric OSA is also associated with cardiovascular dysfunction and deleterious changes in metabolism. In addition to obesity [4] and increased insulin resistance [11], cardiovascular dysfunction includes elevated blood pressure [12], increased systemic proinflammatory cytokines [13,14,15], cardiac left ventricular hypertrophy [16], and endothelial dysfunction [17]. These vascular, metabolic, and cognitive manifestations of OSA during a child’s developmental period can have major consequences later in life and are associated with chronic adult disease such as hypertension [12], diabetes mellitus [18,19], atherosclerosis, and myocardial infarction [20] (Figure 1). These outcomes from more than a decade of research underscore that pediatric OSA is a major risk factor for severe health consequences in adulthood.

Clinical and experimental evidence suggest that a hallmark of OSA, chronic intermittent hypoxia (CIH), is a major contributor to the deleterious consequences of OSA. Cyclical exposure to hypoxia stimulates sympathetic nerve activity [21], generates reactive oxidative species (ROS), and stabilizes hypoxia inducible factor 1 (HIF-1) (reviewed in [22]). HIF is a transcription factor involved in oxygen homeostasis and the regulation of various adaptive responses to hypoxia, including angiogenesis, metabolic reprogramming, and cell survival [23,24]. A recent study found that patients with OSA have chronic stabilization of the oxygen-labile HIF-1⍺ subunit in serum, and that the serum levels directly correlate with the number of oxygen desaturations during sleep [25]. Animal models of OSA have shown that endothelial dysfunction, vascular remodeling, systemic and pulmonary arterial hypertension, and heart failure can develop in response to CIH [21,26,27,28,29,30] and that HIF-1 stabilization is critical for eliciting many of these responses [31,32]. Many of the hypoxia-mediated perturbations of gene expression by epigenetic mechanisms are attributed to these OSA comorbidities (Figure 1). Such heritable, but reversible, epigenetic alterations, including DNA methylation, histone modifications, and the activation of long- and short- non-coding RNAs (lncRNA and miRNA, respectively), have been observed in response to hypoxia and CIH [33,34,35,36]. Importantly, drugs targeting the enzymes that catalyze these dynamic modifications are used for clinical management of some cancers [37] and are in clinical trials for neurological disorders and cardiovascular diseases (reviewed in [38]). Such drugs, or drugs having similar targets, may also benefit pediatric OSA patients by lowering their risk of chronic adult disease; however, there is limited information about the associations between pediatric OSA and epigenetic alterations. A goal of this review is to highlight what is currently known about those associations and to emphasize the importance of epigenetic changes as both modulators of chronic disease and potential druggable therapeutic targets.

## 2. Current Treatments for Pediatric OSA

The most common treatment for OSA in children is adenoidectomy and tonsillectomy (A&T). A&T has highly variable efficacy in treating OSA, and results have shown that the effects of the surgery may only be temporary [39,40,41]. Continuous positive airway pressure, or CPAP, which is widely used to treat adult OSA, improves vigilance and cognitive function, reduces insulin resistance, and is moderately effective in lowering blood pressure in adults with OSA and refractory hypertension. However, the use of CPAP was not found to be associated with reduced risks of cardiovascular disease (CVD), diabetes mellitus, or death for patients with OSA in recent randomized clinical trials [42,43,44,45,46]. Additionally, adherence to CPAP can be difficult for adults, and even more so in children, especially those with behavioral problems and developmental delays [47]. For children with mild OSA (apnea–hypopnea index (AHI) between 1 and 5), high-potency corticosteroids applied intranasally has led to mild improvements in AHI and blood oxygen levels [48]. These steroids have also been shown to reduce the secretion of inflammatory cytokines IL-6, IL-8, and TNF-⍺, which may reduce increased systemic inflammation in children with OSA [10]. Nevertheless, there is a need for more targeted therapeutic treatments as little is known about the longitudinal effects of pediatric OSA and whether current treatments could prevent any long-term consequences of CIH.

## 3. Epigenetic Mechanisms in OSA 

The epigenome harbors important clues regarding the molecular mechanisms underlying human adaptation or maladaptation to stimuli [49,50,51]. In recent years, the field of epigenetics has gained substantial interest as a potential mechanism underlying the etiology and phenotypic variation of multiple diseases [52,53,54,55]. Epigenetic mechanisms that modulate gene regulation include DNA methylation, histone post translational modifications, and noncoding RNAs, such as microRNAs and long noncoding RNAs. All of these gene regulators are influenced by the environment and are likely to have an important role in the pediatric basis of adult disease susceptibility [56]. These epigenetic modifications alter both the chromatin organization and accessibility of genes for transcription factor binding thereby modifying their expression and the expression of gene-related products. Chromatin organization is intimately linked to varied states experienced by any cell in its lifetime, whereby many chromatin changes occur during development in mammals [57,58], in embryonic stem cells transitioning to a differentiated state in vitro [59], and in various diseases [60]. For example, epigenetic alterations have been associated with hypoxia in cancer and a highly complex hypoxia–epigenetic interaction is observed during carcinogenesis and tumor progression [61]. Given the importance of epigenetics in influencing cell functions, a better understanding of both normal and abnormal epigenetic processes will provide deeper insight into the acute and chronic effects of pediatric OSA and may reveal potential new therapies involving epigenetic mechanisms to prevent those effects (Figure 1). The following sections briefly explain the three primary epigenetic regulatory mechanisms (DNA methylation, histone modifications, and noncoding RNAs) and discuss their impact on long-term health during pediatric OSA.

### 3.1. DNA Methylation

During the past decade, investigations of epigenetic modification in OSA have become more frequent and largely focus on DNA methylation in circulating leukocytes. DNA methylation is a heritable epigenetic mark involving the covalent transfer of a methyl group to the C-5 position of the cytosine ring of DNA. The mechanism by which DNA methylation regulates gene expression involves blocking the binding of transcription factors to DNA and the recruitment of proteins containing a methylated CpG-binding domain to inhibit gene expression. DNA methylation is catalyzed by DNA methyltransferases (DMNTs). DNMT1 is generally responsible for maintenance of methylation, while DNMT3a and DNMT3b perform de novo methylation [62]. Depending on the DNA sequence methylated, hypermethylation and hypomethylation can have either an activating or suppressive effect on gene expression, although hypermethylation is generally considered repressive. 

*Clinical*—Although there are limited clinical studies of methylation patterns in pediatric OSA patients, important patterns have been identified that are relevant for inflammation, endothelial dysfunction, and CVD. To determine if epigenetic changes of inflammatory genes are associated with divergent inflammatory phenotypes in children with OSA, one group performed methyl quantitative PCR array assays followed by pyrosequencing of genomic DNA extracted from blood samples of pediatric OSA patients. They found reduced expression and hypermethylation at intron 1 of Forkhead Box P3 (*FOXP3*), a gene critically important in regulation of the Th1 and Th2 cytokine balance [63]. Methylation of the *FOXP3* gene was directly correlated with OSA severity, as measured by AHI. This led to the postulation that during pediatric OSA, *FOXP3* methylation suppresses FOXP3 expression, causing an imbalance of the Th1/Th2 cytokines. Conversely, a recent study investigating the development of subclinical atherosclerosis in adult patients with severe OSA found that plasma FOXP3 expression and *FOXP3* intron 1 methylation was no different than that of control patients regardless of C-reactive protein expression [64]. These divergent data demonstrate the probable and important role of developmental maturation in disease manifestation and progression. 

It was previously shown that expression of the gene encoding endothelial nitric oxide synthase (*NOS3* or *eNOS*) is highly regulated by epigenetic mechanisms including both CpG methylation and histone modifications [65,66], and that these modifications at birth have been associated with bone density and obesity later in childhood [65,67]. These data motivated another study aimed at investigating endothelial dysfunction in children with OSA. In that study, targeted pyrosequencing of *NOS3* was used to identify hypermethylation at the core promoter region of the gene, which was correlated with reduced NOS3 activity and increased peripheral vascular dysfunction in children with OSA [17]. 

*Preclinical*—There is increasing awareness that environmental factors during prenatal and early postnatal periods influence developmental programming of homeostatic mechanisms that profoundly impact susceptibility to disease. Accordingly, Nanduri et al. exposed neonatal rats to CIH as a preclinical model to phenocopy OSA and investigated their cardiorespiratory function as adults. They found that the adult rats which were exposed to CIH as neonates had carotid body hypoxic hypersensitivity that caused irregular breathing, apnea, and hypertension [68]. Enhanced carotid body hypoxic sensitivity was associated with elevated oxidative stress, decreased expression of genes encoding antioxidant enzymes, and increased pro-oxidant enzymes. Decreased expression of the Sod2 gene in the carotid body, which encodes the antioxidant enzyme superoxide dismutase 2, was associated with DNA hypermethylation of a CpG dinucleotide, assessed by qPCR, close to the transcription start site of that gene. Importantly, treating neonatal rats with decitabine, an inhibitor of DNA methylation, during CIH exposure prevented the oxidative stress, enhanced carotid body hypoxic sensitivity, and reduced autonomic dysfunction that was observed in untreated neonatal rats [68]. These findings underscore a role for epigenetic regulation of the genome in mediating neonatal programming of hypoxic sensitivity that is maintained into adulthood, and present evidence for targeted therapy of epigenetic marks for re-programming.

### 3.2. Histone Modifications

The study of histone post translational modifications (PTMs) during sleep apnea is an emerging field. Understanding the global effects of individual histone modifications that culminate to create a “histone code” has been an important goal for determining the role these PTMs have in modulating both acute and chronic chromatin accessibility in health and disease. In response to periods of hypoxia during OSA, local histone PTMs can function to either activate or suppress the transcription of genes that ultimately results in a sustained response to the hypoxic periods. There are limited studies reporting histone PTMs in adult OSA (reviewed in [69,70]), and to our knowledge, there are no reports of PTMs in the pediatric OSA field. The following sections will briefly discuss some of the current data from adult OSA studies to emphasize the importance of these modifications in the pathogenesis of OSA-induced morbidity, and this gap of knowledge in the pediatric population. Although histone modifications are imposed by methylation, acetylation, ubiquitylation, phosphorylation, and sumoylation of various amino acid residues [71], methylation and acetylation of lysine residues are the most studied in OSA and will be discussed in the following sections. 

#### 3.2.1. Histone Methylation

Histone methylation is the addition of methyl groups to histone tail residues. Methylation of histone tails is governed by positive and negative regulators, and each mark can have an activating or suppressive effect on transcription depending on the amino acid residue. Achieved through the action of histone methyltransferases or histone demethylases, histone lysine methylation can occur in three different states: mono-, di- or trimethylation [72]. Typically, the marks that activate gene transcription include the di- or trimethylation at the H3K4, H3K36, and H3K79 sites [73,74,75]. While H3K4 trimethylation acts on enhancer and promoter regions, H3K36 and H3K79 act over gene bodies [73,74,75]. H3K9 and H3K27 methylations are typically considered repressive [76]. 

*Adult preclinical histone methylation*—While our understanding of activating or repressive histone methylation marks has grown over the past decades, specific methylation marks associated with pediatric sleep apnea have yet to be elucidated. Accumulating evidence from adult OSA studies confirm the presence and importance of these marks in response to CIH. In a recent study of adult mice exposed to CIH, macrophages isolated from the aorta had significant accumulation of the repressive histone mark H3K27me3 associated with anti-inflammatory and glutathione redox pathway member genes that protect against atherosclerosis, such as peroxisome proliferator-activated receptor/retinoid X receptor and liver X receptor/retinoid X receptor [77]. These epigenetic changes occurred in parallel with recruitment of macrophages to the aortic wall and the triggering of atherogenesis. These results indicate that histone modification-mediated activation of the oxidative stress and inflammatory pathways may be involved in the establishment of CIH-induced endothelial dysfunction, atherosclerosis, and aortic remodeling in OSA. 

#### 3.2.2. Histone Acetylation

Histone acetylation occurs when an acetyl group is transferred onto the lysine residue of a histone tail and generally contributes to increased accessibility of transcriptional machinery to chromatin. Histone acetylation is achieved through the catalytic enzymes known as histone acetyltransferases (HATs). Conversely, histone deacetylases (HDACs) function to decrease histone acetylation, thereby reducing accessibility of chromatin to transcriptional regulators. HDACs are stratified into four different classes based on their structure and function [78]. HDAC expression and subsequent histone acetylation patterns have been studied minimally in adult OSA, and results suggest a direct correlation with disease phenotype. 

*Adult clinical histone acetylation*—In adult patients with moderately severe OSA, expression of Sirtuin 1 (SIRT1), a Class III HDAC, was found to be reduced in peripheral blood cells. SIRT1 has an important regulatory role over transcriptional regulators such as p53, NF-kB, NOS3, and FOXO. Moreover, disruption of SIRT function has been implicated in metabolic and CVDs. Following 3 months of CPAP, expression and activity of SIRT1, as well as plasma nitric oxide derivative, were restored [79]. This result highlights both the potential plasticity of inducing and removing histone acetylation and the significant role of acetylation in pathogenesis. 

In a separate study utilizing adipose tissue from OSA patients, a number of gene sets were upregulated compared to control, representing the pro-inflammatory NF-_K_B pathway and the proteolytic ubiquitin/proteasome module [80]. Further network analysis of these pathways suggested HDAC2 as a hub protein for this dysregulation [81]. Interestingly, HDAC2 has previously been shown to physically interact with FOXO3a and regulate FOXO3a-dependent gene transcription pathways such as inflammation, apoptosis, and the response to oxidative stress [82,83]. 

*Adult preclinical histone acetylation*—To identify the mechanism underlying accelerated atherogenesis in OSA patients, Cortese and colleagues exposed mice to CIH and found increased accumulation and proliferation of pro-inflammatory macrophages expressing CD36 in the aorta [77]. Assessed by ChIP-seq, the macrophages had significant accumulation of the active histone mark H3K9Ac associated with genes of pro-inflammatory and oxidative stress signaling pathway members, including HIF-1, p53, NF-_K_B, tumor growth factor-β, FOXO4, and IL-6 [77]. Importantly, discontinuation of CIH did not elicit significant improvements in aortic wall macrophage phenotype, suggesting long-term CIH-induced changes may not be reversible solely by cessation of cyclic hypoxia. 

A recent elegant study using rat pheochromocytoma (PC)-12 cells, mice, and rats, found that HDAC5 modulates CIH-induced autonomic dysfunction. In this study, Wang et al. showed that CIH reduces HDAC activity by promoting proteasomal degradation of HDAC3 and HDAC5, contributing to increased histone 3 acetylation as well as increased HIF-1 stabilization through lysine acetylation of the HIF-1⍺ subunit [84]. The resulting increased HIF-1 transcriptional activity prompted sympathetic nerve activation and hypertension in mice and rats. Other common acetylation marks that are activated at enhancer and promoter regions include H3K27Ac [85] and H4K16Ac [86]; however, there is a general lack of knowledge regarding how these marks contribute to adult or pediatric obstructive sleep apnea. 

### 3.3. Noncoding RNAs

Noncoding RNAs consist of microRNAs (miRNAs) and long non-coding RNAs (lncRNAs). Over the past decade, the understanding of the role of RNA has shifted; there is increasing evidence that only 1–2% of RNA codes for proteins [87,88], while non-coding RNA has a predominant and essential role in modulating gene expression. 

#### 3.3.1. miRNA

MicroRNAs are single-stranded RNAs that are 18–22 nucleotides in length and negatively regulate gene expression at the post transcriptional level by binding to mRNA, which can then lead to translational repression [89,90]. Considered an ideal biomarker in the era of precision medicine, miRNAs have the capacity to regulate most protein encoding genes; thus, the up- or downregulation of certain miRNAs can have downstream effects on target genes. MicroRNAs have been studied considerably in adult OSA, but comparatively little is known about miRNAs in pediatric OSA. To date, only two clinical studies have investigated miRNAs in children with OSA. These studies are discussed below.

*Clinical miRNA*—Since cardiovascular effects such as elevated blood pressure and vascular abnormalities are associated with OSA, one group investigated whether circulating exosomal miRNAs of children with OSA differentiate based on endothelial functional status. They used microarray expression data to identify reduced miRNA-630 expression in plasma exosomes of children aged 4–12 with OSA and endothelial dysfunction, an early risk factor for atherosclerosis and CVD [91]. Importantly, mimic miR-630 administered to human endothelial cells lacking miRNA-630 in vitro restored indices of endothelial cell function. Additional gene target discovery experiments further revealed that miRNA-630 regulates 416 gene targets that include the NRF2, AMP kinase, and tight junction pathways. As such, the diagnosis and treatment of OSA associated CVD, before the onset of irreversible disease, is essential. 

Elevated serum miRNA-92a has previously been found in patients with coronary artery disease and is known to elicit endothelial dysfunction and early onset CVD [92,93]. In an effort for earlier diagnosis of CVD in OSA, another group tested for elevated miR-92a in children and adults with OSA to determine its potential to serve as a biomarker for childhood diagnosis of OSA associated CVD. They found upregulation of miR-92a in the serum of both adults and children diagnosed with OSA, with expression levels correlating to disease severity [94]. These data complement the findings from adult OSA patient studies that have identified over 100 dysregulated plasma miRs with target genes highly enriched in metabolic signaling [95], CVDs, inflammation and cancer pathways [90].

#### 3.3.2. LncRNA

Long non-coding RNAs consist of RNA strands that are longer than 200 nucleotides. These lncRNAs can be up- or downregulated in disease progression and can modulate gene expression through a variety of mechanisms such as altering chromatin recruitment or chromatin modifiers and can suppress transcription initiation by interfering with RNA polymerase [96]. LncRNAs have been studied in the development of obesity, with implications in the process of adipogenesis through the modulation of the gene expression of cell cycle marker genes including cyclin B, D, and E [97]. LncRNAs also have a key function in mitigating myocardial infarction and myocardial ischemia–reperfusion injury, and heart failure (reviewed in [98]). There are numerous publications that describe the role of lncRNAs in metabolic syndromes such as obesity, type 2 diabetes, CVD, and pulmonary hypertension, all of which are comorbidities of sleep apnea. However, only a few published studies have investigated the role of lncRNAs in the genesis and progression of OSA in adults and more importantly, in children.

*Clinical lncRNA*—Associations between decreased glucocorticoid receptor ⍺ (GR⍺) expression and inflammation in the adenoids of children with OSA have been identified. In an effort to describe the role and mechanism of GR⍺ in OSA pathogenesis, Zhou et al. [99] found lncRNA XIST as closely associated with GR⍺, and its increased expression in adenoids of pediatric OSA patients. Using NP69 cells, lncRNA XIST reduced expression of GR⍺ which then significantly increased inflammatory cytokines, including interleukin (IL)-8, TNFa, IL-6, and IL-1β. These data link elevated lncRNA XIST expression in pediatric OSA patients to increased inflammation via downregulation of GR⍺. To our knowledge, this is the only published report to-date of lncRNA expression profiles in children with OSA, despite the abundance of compelling evidence that lncRNA are differentially regulated in adults with OSA [100], in response to cyclic hypoxia, and are linked to CVD. A deeper understanding of how lncRNAs regulate gene expression in pediatric sleep apnea will likely identify promising new therapeutic targets that could prevent the severe adult complications and disease that originate from childhood OSA.

## 4. Epigenetic Therapies

Although DNA methylation and histone modifications are endogenously reversible, these modifications that develop in childhood due to sleep apnea are likely to have a long-lasting effect on the cardiovascular system that will continue with the child into adulthood, as is evidenced by preclinical studies of DNA methylation [68]. The plasticity of chromatin regulation makes targeting the enzymatic machinery, or the reversible alterations themselves, an attractive strategy for therapeutic intervention. In fact, an increasing number of small molecule inhibitors designed to counteract a variety of epigenetic regulators are currently under development or are already used clinically. Epigenetic biomarkers and targeted epigenetic therapies such as the histone deacetylase (HDAC) inhibitors Vorinostat, Panobinostat, Romidepsin and Belinostat, are approved for certain lymphomas and myeloma. Cardiovascular studies using HDAC inhibitors have shown promise in pre-clinical management of cardiac hypertrophy, heart failure, oxidative stress, hypertension and cardiac fibrosis [101,102,103,104]. Recently, Givinostat, a clinical-stage inhibitor of HDAC catalytic activity was shown efficacious in two distinct murine models of diastolic dysfunction with preserved ejection fraction, by relieving impaired cardiac myofibril relaxation [105]. HDAC inhibitors have also been shown to have a dose-dependent anti-inflammatory effect, decreasing inflammatory cytokines such as TNF-⍺ and IFN-β in models of irritable bowel disease [106]. Determining the extent of epigenetic alterations that occur in response to pediatric OSA and the mechanisms by which they influence the neonatal basis of disease susceptibility and progression is required for development of new epigenetic targeted treatments for OSA in neonates and adults.

## 5. Concluding Remarks

Pediatric OSA has significant negative effects on behavior and health in children, including depression, failure to thrive, neurocognitive impairment, excessive daytime sleepiness, increased risk for systemic and pulmonary hypertension, and behavioral issues suggestive of attention-deficit/hyperactivity disorder [107,108]. Importantly, pediatric OSA is strongly associated with an increased risk for a variety of end-organ injury and dysfunction in adults, such as obesity and diabetes, accelerated atherosclerosis, and endothelial dysfunction; syndromes that impose both immediate and long-term morbidities resulting in high healthcare costs [3,40]. Deeper investigations of epigenetic regulators that interact to modify gene expression will provide not only detailed mechanistic understanding of disease progression but also new insights into the fetal basis of adult disease susceptibility. Building upon the evidence that DNA methylation, post-translational histone modifications, and noncoding RNAs all modulate cellular reprogramming due to exposure to hypoxia, a thorough understanding of how these chromatin modifications work synergistically will be crucial for developing the most effective targeted treatments. The promise is that such treatments will target the most fundamental underpinnings of chronic disease by blunting the deleterious epigenetic programming that occurs during pediatric OSA. Gaining a broader understanding of how epigenetic regulators are modulated during a child’s developmental period and are dysregulated due to cyclic hypoxia is an extremely important direction for the future of pediatric OSA research.
